# Dr. Charles W. Carrier

**Published:** 1914-06

**Authors:** 


Dr. Charles W. Carrier, Buffalo, 1862; died at his home in Troy, Pa., March 24, aged 73.

